# Non-pharmacological interventions for side effects of antineoplastic chemotherapy prioritized by patients: systematic review

**DOI:** 10.15649/cuidarte.3612

**Published:** 2024-10-11

**Authors:** María Elizabeth Gómez-Neva, Edwin Pulido-Ramirez, Leidy Johana Ibañez-Rodriguez, Oscar Caroprese, Adriana Buitrago-Lopez

**Affiliations:** 1 Pontificia Universidad Javeriana, Facultad de Enfermería. Bogotá, Colombia. E-mail: m.gomezn@javeriana.edu.co Pontificia Universidad Javeriana Pontificia Universidad Javeriana Facultad de Enfermería Bogotá Colombia m.gomezn@javeriana.edu.co; 2 Pontificia Universidad Javeriana. Departamento de Epidemiología Clínica y Bioestadística, Facultad de Medicina. Bogotá, Colombia. E-mail: pulidoedwin@javeriana.edu.co Pontificia Universidad Javeriana Pontificia Universidad Javeriana Departamento de Epidemiología Clínica y Bioestadística Facultad de Medicina Bogotá Colombia pulidoedwin@javeriana.edu.co; 3 Hospital Universitario San Ignacio. Bogotá, Colombia. E-mail: leidy.ibanez@javeriana.edu.co Hospital Universitario San Ignacio Bogotá Colombia leidy.ibanez@javeriana.edu.co; 4 Subred Integrada de Servicios de Salud Norte. Bogotá, Colombia. E-mail: ocaropresse@g mail.com Subred Integrada de Servicios de Salud Norte Bogotá Colombia ocaropresse@g mail.com; 5 Pontificia Universidad Javeriana, Departamento de Epidemiología Clínica y Bioestadística, Facultad de Medicina. Bogotá, Colombia. E-mail: buitragod@javeriana.edu.co Pontificia Universidad Javeriana Pontificia Universidad Javeriana Departamento de Epidemiología Clínica y Bioestadística Facultad de Medicina Bogotá Colombia buitragod@javeriana.edu.co

**Keywords:** Complementary Therapies, Drug-Related Side Effects and Adverse Reactions, Integrative Oncology, Signs and Symptoms, Terapias Complementarias, Efectos Colaterales y Reacciones Adversas Relacionados con Medicamentos, Oncología integrativa, Signos y Síntomas, Terapias Complementares, Efeitos Colaterais e Reações Adversas Relacionados a Medicamentos, Oncologia Integrativa, Sinais e Sintomas

## Abstract

**Introduction:**

Different non-pharmacological interventions have been studied to manage symptoms derived from chemotherapy, but their effectiveness is unknown.

**Objective:**

To describe non-pharmacological interventions for managing symptoms secondary to antineoplastic chemotherapy in adults.

**Materials and Methods:**

Systematic review of analytical experimental and observational studies (2021 to 2023). The studies were selected, and data was extracted in parallel. Discrepancies were resolved with a third reviewer. The risk of bias was assessed using the Risk of Bias (RoB) tool and The Newcastle-Ottawa Scale (NOS). The literature was synthesized descriptively based on prioritized outcomes.

**Results:**

The prioritized outcomes were neutropenia, pain, neuropathy, nausea, vomiting, alopecia, anorexia, and sleep disorders. Out of 7520 references found, 62 were included for analysis. Acupressure showed a possible effect in controlling symptoms such as nausea and vomiting. The intervention with cold on the scalp showed differences in the stages of alopecia severity. Other interventions showed heterogeneity.

**Discussion:**

Non-pharmacological interventions have been widely described in observational and experimental studies in the control of side effects of chemotherapy; however, there is homogeneity and a high risk of bias.

**Conclusion:**

Acupressure, muscle massage, music therapy, foot baths, and other interventions have been studied for nausea, vomiting, sleep disorders, neutropenia, alopecia, anorexia, pain, and neuropathy as secondary symptoms prioritized by patients. It is necessary to standardize both the interventions and how measure the outcomes.

## Resumen.

**Introducción:**

Diferentes intervenciones no farmacológicas se han estudiado para manejar los síntomas derivados de la quimioterapia, pero se desconoce su efectividad.

**Objetivo:**

Describir las intervenciones no farmacológicas para el manejo de síntomas secundarios a la quimioterapia antineoplásicaenadultos.

**MaterialesyMétodos:**

Revisiónsistemáticadeestudiosexperimentales y observacionales analíticos (2021 a 2023). La selección de estudios y extracción de datos se realizó de forma paralela. Las discrepancias se resolvieron con un tercer revisor. Se evaluó el riesgo de sesgo con las herramientas Risk Of Bias (RoB) y The Newcastle-Ottawa Scale (NOS). La síntesis de la literatura se realizó de forma descriptiva por desenlace priorizado.

**Resultados:**

Los desenlaces priorizados fueron neutropenia, dolor, neuropatía, náuseas, vomito, alopecia, anorexia y desordenes del sueño. Se encontraron 7520 referencias, 62 incluidas para el análisis. La acupresión mostró un posible efecto en el control de síntomas como las náuseas y vomito. La intervención con frio en el cuero cabelludo mostro diferencias en los estadios de la severidad de alopecia. Las otras intervenciones mostraron heterogeneidad.

**Discusión:**

Las intervenciones no farmacológicas han sido ampliamente descritas en estudios observaciones y experimentales en el control de efecto secundarios a la quimioterapia, sin embargo, existe homogeneidad, y alto riesgo de sesgo.

**Conclusión:**

Acupresión, masaje muscular, musicoterapia, baño de pies entre otros son las intervenciones que se han estudiado para náuseas, vomito, desordenes del sueño, neutropenia, alopecia, anorexia, dolor y neuropatía como síntomas secundarios priorizados por pacientes. Se requiere estandarizar tanto las intervenciones como la forma de medición de los desenlaces.

## Resumo.

**Introdução:**

Diferentes intervenções não farmacológicas têm sido estudadas para o manejo dos sintomas decorrentes da quimioterapia, mas sua eficácia é desconhecida.

**Objetivo:**

Descrever intervenções não farmacológicas para o manejo dos sintomas secundários à quimioterapia antineoplásica em adultos.

**Materiais e Métodos:**

Revisão sistemática de estudos analíticos experimentais e observacionais (2021 a 2023). A seleção dos estudos e a extração dos dados foram realizadas paralelamente. As discrepâncias foram resolvidas com um terceiro revisor. O risco de viés foi avaliado por meio das ferramentas Risk Of Bias (RoB) e Newcastle-Ottawa Scale (NOS). A síntese da literatura foi realizada de forma descritiva por desfecho priorizado.

**Resultados:**

Os desfechos priorizados foram neutropenia, dor, neuropatia, náuseas, vômitos, alopecia, anorexia e distúrbios do sono. Foram encontradas 7.520 referências, 62 incluídas para análise. A acupressão mostrou possível efeito no controle de sintomas como náuseas e vômitos. A intervenção fria no couro cabeludo mostrou diferenças nos estágios de gravidade da alopecia. As demais intervenções apresentaram heterogeneidade.

**Discussão:**

Intervenções não farmacológicas têm sido amplamente descritas em estudos observacionais e experimentais no controle dos efeitos colaterais da quimioterapia, porém há homogeneidade e alto risco de viés.

**Conclusão:**

Acupressão, massagem muscular, musicoterapia, escalda-pés, entre outras, são as intervenções que têm sido estudadas para náuseas, vômitos, distúrbios do sono, neutropenia, alopecia, anorexia, dor e neuropatia como sintomas secundários priorizados pelos pacientes. É necessário padronizar tanto as intervenções quanto a forma de medir os resultados.

## Introduction

In 2020, Globocan reported 19,292,789 new cancer cases worldwide^1^. Specific treatment regimens have been studied for each type of disease, with chemotherapy being the main intervention^2^. The incidence of side effects is reported to be 70-80% due to the involvement of rapidly growing cells^3^. There is evidence ofside effects such as nausea, vomiting, alopecia, mucositis, fatigue, constipation, neutropenia, and mood changes, which affect a person's quality of life^4^^, ^^5^. Treatment plans include medications to control these symptoms; however, these medications can trigger other secondary symptoms that further impact the quality of life^6^.

Integrative oncology, in coordination with evidence-based complementary therapies and conventional cancer care, improves patients' quality of life and clinical outcomes. This orientation empowers patients' participation in their treatment^7^. It has been reported that approximately 50% of cancer patients use complementary and alternative medicine (CAM), and in patients with advanced disease, the prevalence of CAM use can reach 100%^7^.

The evidence shows a wide varietyofnon-pharmacological interventions, which presentsa challenge to the caregiver when seeking symptom control. This process involves balancing pharmacological treatment, complementation with non-pharmacological interventions, and individual preferences^8^. This review aims to synthesize the existing evidence on non-pharmacological interventions to control the side effects of chemotherapy, as prioritized by patients and healthcare professionals.

## Materials and Methods

The protocol was published in the International Prospective Register of Systematic Reviews (PROSPERO CRD4202017212) and conducted according to the Preferred Reporting Items for Systematic Reviews and Meta-analyses (PRISMA, 2009) ^9^ guidelines; the analysis database was stored in Mendeley Data^10^. We included randomized clinical trials (RCTs) and longitudinal analytic observational studies conducted in adults with cancer undergoing treatment that described non-pharmacological interventions to control chemotherapy-related side effects. Studies were only included in the review if the nonpharmacological interventions were delivered by trained personnel. Descriptive studies, cost-effectiveness studies, conference proceedings, systematic reviews, meta-analyses, clinical practice guidelines, letters to the editor, or studies with unanalyzable data or without reported measures of effect, animal studies, or studies in pregnant women were excluded.

### Outcome selection and prioritization

**Figure 1:**
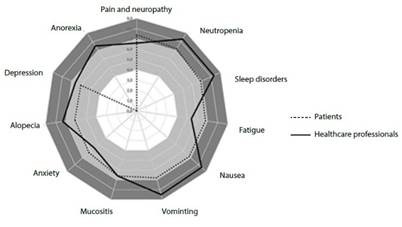
Prioritization of symptoms secondary to chemotherapy by healthcare professionals and cancer patients

The outcomes were prioritized according to the preferences of patients and health professionals at the time of making a decision about an intervention, including the list described in the literature^3^^, ^^11^. Ten cancer experts and chemotherapy patients from a university hospital oncology department were independently asked to prioritize each side effect on a scale of 1 to 9, with 7 to 9 being critical, 4 to 6 being important, and 1 to 3 being of limited importance (according to the GRADE approach^12^). For this review, outcomes with scores greater than 8 were included (Figure 1).

### Search strategy

The electronic databases PubMed/MEDLINE, Ovid Embase, LILACS/Bireme, The Cochrane Library, and Epistemonikos were searched from March 2021 to May 2023. University repositories and reference lists of included studies were also searched. Authors and clinical experts in cancer were also contacted to inquire about possible published studies in this area. The search algorithm was developed using free search terms and the Medical Subject Headings (MeSH) (Table 1).

**Table 1:** Search strategies used in PubMed, Embase, and LILACS

Database	Search strategy
PubMed	((((carcinoma chemotherapy OR chemotherapy OR "Chemotherapy, Cancer, Regional Perfusion"[Mesh] OR "Chemotherapy, Adjuvant"[Mesh]) OR "Chemotherapy, Cancer, Regional Perfusion"[Mesh] OR "Chemotherapy, Adjuvant"[Mesh], carcinoma chemotherapy, carcinoma chemotherapy, antineoplastic drugs, "Antineoplastic Agents"[Mesh] OR "Antineoplastic Agents" [Pharmacological Action]) NOT (Child[Mesh] OR oncology pediatric OR pediatric OR child* OR children)) AND (("Oncology Nursing"[Mesh], OR nursing practices, cancer nursing, palliative care nurse, nursing care, nursing interventions, nursing intervention, "Oncology Service, Hospital"[Mesh] OR "Oncology Nursing"[Mesh], "Hospice and Palliative Care Nursing"[Mesh] OR "Oncology Nursing"[Mesh] OR "Nursing Care"[Mesh] OR "Patient Care Planning"[Mesh] OR home care) NOT ((non-pharmacological intervention) OR (non-pharmacological treatment) OR (non-pharmaco*)))) AND ((((((alopecia) OR (((Sleep Wake Disorders) AND (Sleep Disorders, Intrinsic)) AND (((neutropenia) AND ((nausea) AND (vomiting))) AND ((anorexia))))) OR (((neutropenia) AND ((nausea) AND (vomiting))) AND ((anorexia)))) OR ("Metabolic Side Effects of Drugs and Substances"[Mesh] OR "Drug-Related Side Effects and Adverse Reac-tions"[Mesh] OR secondary side effects)) OR (((Peripheral Nervous System Diseas-es) AND (Peripheral Neuropathies)) AND (pain))) OR ("Metabolic Side Effects of Drugs and Substances"[Mesh] OR "Drug-Related Side Effects "[Mesh] OR second-ary side effects))
Embase	#11 AND (2020:py OR 2021:py OR 2022:py OR 2023:py) AND 'vomiting'/dm AND ('clinical article'/de OR 'clinical study'/de OR 'clinical trial'/de OR 'clinical trial topic'/de OR 'cohort analysis'/de OR 'controlled clinical trial'/de OR 'controlled study'/de OR 'cross sectional study'/de OR 'double blind procedure'/de OR 'evidence based medicine'/de OR 'evidence based practice'/de OR 'human'/de OR 'human ex-periment'/de OR 'intervention study'/de OR 'interview'/ de OR 'longitudinal study'/de OR 'major clinical study'/de OR 'multicenter study'/de OR 'normal human'/de OR 'observational study'/de OR 'open study'/de OR 'pilot study'/de OR 'prospective study'/de OR 'randomized controlled trial'/de OR 'randomized controlled trial top-ic'/de) AND ([adult]/lim OR [aged]/lim OR [middle aged]/lim OR [very elderly]/lim OR [young adult]/lim) AND ('article'/it OR 'article in press'/it)
LILACS	(tw:("palliative care nursing" OR "nursing interventions" OR "nursing care" OR "cancer nursing" OR "Enfermagem Oncológica" OR "Hospice and Palliative Care Nursing")) AND (tw:("Efeitos Colaterais e Reações Adversas Relacionados a Medicamentos" OR "efeitos adversos" OR "side effect" OR "adverse effect" OR "adverse events" OR "drug side effect" AND "Tratamento Farmacológico" OR "Tratamento Farmacológico" OR "Antineoplásicos" OR "Tratamento Farmacológico" OR "Antineoplásicos" OR "Quimioterapia Adjuvante"))

### Study selection and data extraction

Two groups of reviewers (Group 1 - MEG-N and ABP; Group 2 - LI/EP and OC) independently screened references found by title and abstract according to the RAYYAN eligibility criteria for systematic reviews^13^. Two reviewers read full texts for final inclusion. Disagreements were resolved with the assistance of a third reviewer (AB-L). A matrix was created in Microsoft Excel® in which two independent reviewers entered data including authors, year of publication, study’s country of origin, sex, cancer diagnosis, comorbidities, sample size, study population, non-pharmacological intervention used, and measure of the effect in both the experimental and control groups. The authors were contacted to request information on missing data.

### Risk of bias assessment

Figures 2A**,**B**,**C**,**D**,**E**,** and F show the graphical visualization of the risk of bias for experimental studies assessed with the RoB-2 tool^14^. Figure 2G shows the risk of bias assessment for analytic observational cohort studies assessed with the Newcastle-Ottawa Scale (NOS) ^15^ (Figure 2).

**Figure 2A:**
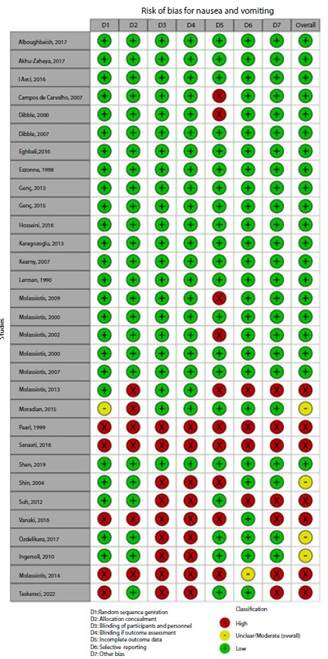
Risk of bias of articles with experimental study design included in the outcome nausea and vomiting

**Figure 2B:**
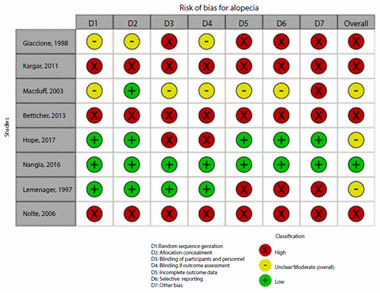
Risk of bias of articles with experimental study design included in the outcome alopecia

**Figure 2C:**
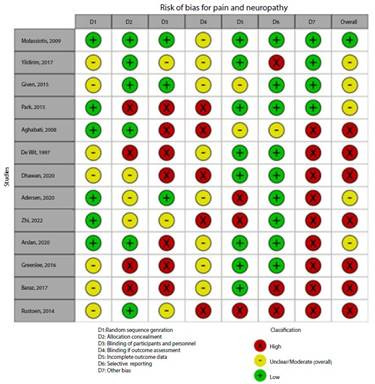
Risk of bias of articles with experimental study design included in the outcomes pain and neuropathy

**Figure 2D:**
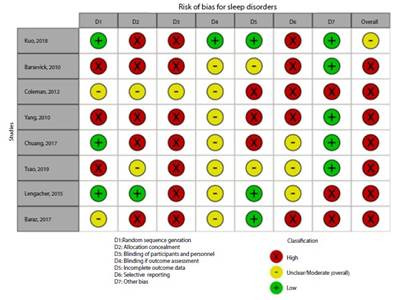
Risk of bias of articles with experimental study design included in the outcome sleep disorder

**Figure 2E:**
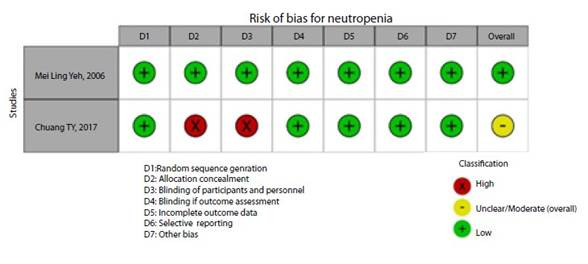
Risk of bias of articles with experimental study design included in the outcome neutropenia

**Figure 2F:**
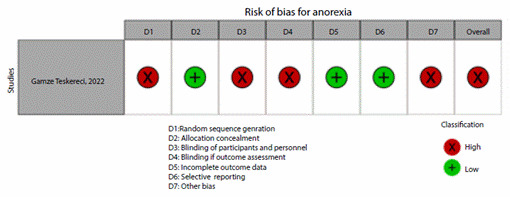
Risk of bias of articles with experimental study design included in the outcome anorexia

**Figure 2G:**
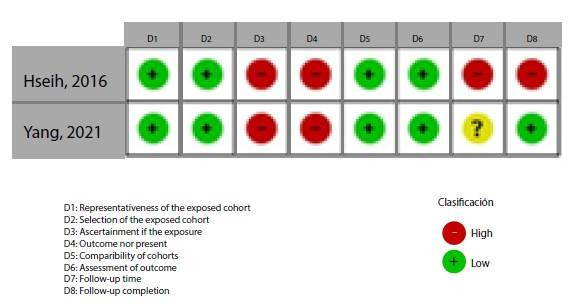
Risk of bias in observational analytical cohort studies

### Synthesis of evidence

Study characteristics were described narratively by outcome. The heterogeneity of the studies was assessed by clinical observation of the population, outcomes and their measurement, and description of the intervention performed^10^.

## Results

**Figure 3:**
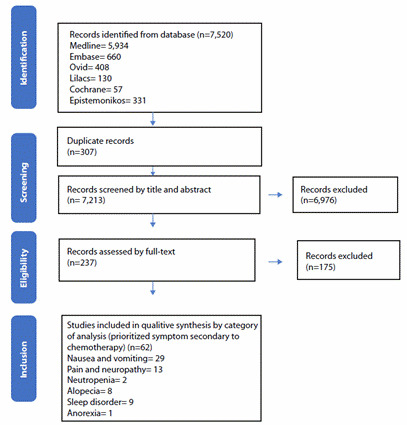
PRISMA description of search findings and study selection

A total of 7,520 references were found, of which 237 were selected for full-text reading. Sixty-two references were included between 1988 and 2023 (Figure 3). Nineteen interventions evaluating 6,613 participants were identified across all studies in the United States, and 4,577 women participated.

### Nausea and vomiting

Twenty-nine references were included; 25(89.21%) are RCTs and 4(13.73%) are quasi-experiments with participants between 16 and 96 years of age. We reviewed 4(12.91%) care and counseling programs, 5(16.14%) muscle relaxation techniques, 4(12.95%) guided relaxation with music therapy and imagery, 2(6.44%) natural drinks, 2(6.43%) therapeutic touch and reflexology, 12(38.70%) acupressure at P6 point, and 2(6.41%) hologram bracelets. Studies on interventions such as acupressure were consistent in affirming that there was improvement before and after the intervention; however, they showed high heterogeneity regarding the types of interventions and scales used to measure nausea and vomiting (Table 2).

### Anorexia

One RCT conducted in Turkey^16^ involving women aged 29 to 69 years with stage II or III gynecological cancer was included. The intervention involved a nursing program based on Jean Watson's theory. Nursing professionals visited and followed up with the participants via telephone for 60 to 120 minutes once a week. Information on symptom management was provided and compared with standard hospital management. The authors assessed changes in appetite using the Chemotherapy Symptom Assessment Scale (C-SAS). They found that the intervention group had a lower mean change in appetite of 1.00 SD (0.61) than the control group of 2.00 SD (1.08). This study had a high risk of bias due to the lack of randomization and blinding.

### Alopecia

Eight studies evaluated non-pharmacological interventions to control alopecia, such as scalp cooling with hypothermic caps, and one study used videos on makeup and wigs. Five studies used WHO criteria to evaluate the effect of scalp cooling on reducing alopecia. The other studies used instruments such as the Dean scale, the Common Terminology Criteria for Adverse Events (CTCAE) version 4.0, and the breast cancer stem cells (BC SCs) to assess the efficacy of the intervention on hair loss. In general, these studies have a high risk of bias, and scalp cooling shows a possible effect on reducing alopecia compared to placebo (Table 3).

### Pain and neuropathy

A total of 1,403 patients, aged 15 to 86 years, were observed in 14 studies. Interventions included educational programs, acupuncture, physical activity, psychological therapies, natural substance applications, massages, and foot baths. Pain and neuropathy were measured using the National Cancer Institute Common Terminology Criteria for Adverse Events (NCI-CTC), Numerical Pain Scale (NPS), the Dutch Language Version of the McGill Pain Questionnaire (MPQ-DLV), and Symptom Experience Scale. Of the total, 6 (42.81%) studies evaluated disease-related pain, and 8 (57.20%) studies evaluated platinum or taxane chemotherapy-related neuropathy (Table 4). These studies have a high risk of bias due to selective reporting of outcomes, lack of concealment, and lack of blinding. Interventions such as home-based care nursing programs and acupuncture were demonstrated to reduce mean pain and neuropathy when comparing pre- and post-intervention measurements.

### Sleep disorders

Nine studies evaluated non-pharmacological interventions to control sleep disorders. Acupressure, telephone follow-up programs, home exercises, relaxation therapies such as foot baths, mindfulness therapies, back massages, and Chinese practices like Chan-Chuang qigong have been studied for their effectiveness in improving sleep quality. However, it is observed that interventions such as acupressure and physical exercise improve sleep quality when comparing intervention groups with post-intervention control groups (Table 5).

### Neutropenia

Two studies analyzed 167 participants diagnosed with neutropenia, defined as a decrease in neutrophils following chemotherapy treatment, and administered Chan-Chuang qigong therapy for 21 minutes over 21 days. This technique includes mind and body relaxation, with white blood cell counts measured before and after the procedure. The studies have a high risk of bias due to the non randomization of participants, but the intervention showed an increase in white blood cell counts after the intervention (Table 6).

**Table 2:** Nonpharmacological interventions: Nausea and vomiting outcome

						Outcome	
Author, year	Design	Population and population size N	Instrument	Intervention	Outcome	Before		After	
						Intervention	Control	Intervention	Control
Nursing intervention programs									
Teskereci, 2022^16^	Randomized clinical trial	Gynecologic cancer N=52	Herth Hope Scale	Nursing program based on Watson's Theory of Human Caring	Nausea severity Mean (SD)			1.0 (0.84)	3.0 (0.75)
Molassioti, 2009^17^	Randomized clinical trial	Colorectal and breast cancer N=164	Chemotherapy Symptom Assessment Scale (C-SAS)	Home nursing care program for symptom management	Nausea severity Mean (SD)			1.0 (0.84)	3.0 (0.75)
Alboughobeish, 2017^18^	Quasi experimental	Different types of cancer		Mobile care program designed by nurses	Vomiting frequency. Mean (SD)	1.8 (1.77)	1.64 (1.84)	0.84 (1.37)	2.48(2.16)
Kearney, 2007^19^	Randomized clinical trial	Lung, colorectal, and breast cancer N= 112	Advanced symptom management system (ASyMS©)	Mobile care program designed by nurses	Severity of vomiting distress. Mean (SD) Severity of nausea distress (SD)			0.51 (0,93) 1.23(1.19)	0.50 (0.81) 1.43 (1.08)
Campos de Carvalho, 2007^20^	Pretest -Posttest	Different types of cancer N=30	Huskisson's visual analog scale	Muscle relaxation therapy	Level of nausea Median (IQR) Level of vomiting. Median (IQR)	6.00 (3.75-7.00) 4.00 (2.00-5.25)		4.50 (3.00-6.00) 2.00 (1.00-3.00)	
Molassioti, 2000^21^	Randomized clinical trial	Breast cancer. N= 8	Morrow assessment of nausea and vomiting (MANE)	Muscle relaxation program	Nausea duration. Hours Vomiting duration. Hours	7 hours 2.75 hours		1.5 hours 1.67 hours	
Lerman, 1990^22^	Randomized clinical trial	Different types of cancer N=96	Emesis Rating Scale	Muscle relaxation techniques	Nausea prevalence N (%)	5(46%)	3(27%)	6(54%)	8(73%)
Sensory distraction techniques									
Ezzonne, 1998^23^	Randomized clinical trial	Bone marrow transplant N= 39	Thermometer shaped visual analog scale	Music therapy	Vomiting episodes. Mean (range)	0.69 (0-4)	1.73 (0-6)	0.94(0-2)	0.31 (0-2)
Hosseini, 2016^24^	Quasi experimental	Breast cancer N=55	Morrow Assessment of Nausea and Vomiting	Image illustration and audio CD	Nausea severity. Mean (SD) Nausea frequency. Mean (SD) Vomiting severity. Mean (SD) Nausea frequency. Mean (SD)	a. 1.91 (1.97) b. 1.67 (0.88) c. 0.48 (0.09) d. 1.10 (0.24)		a. 2.07 (1.63) b. 1.91 (0.63) c. 0.62 (0.05) d. 0.42 (0.05)	
Karagozoglu, 2013^25^	Randomized clinical trial	Lung, gastric, and breast cancer N= 40	Visual Analog Scale (VAS)	Music therapy and visual imagery	Nausea severity. Hours Vomiting severity. Hours Nausea duration. Hours (1-4h) Vomiting duration. Hours (1- 4h)	a. 5 (12.5%) b. 1 (2.5%) c. 5 (12.5%) d. 6 (15%)	a. 4 (10%) b. 2 (5%) c. 8 (20%) d. 7(17.5%)	a. 8 (20%) b. 9 (22.5%) c. 7 (17.5%) d. 8 (20%)	a. 2 (5%) b. 0 c. 8 (20%) d. 9(22.5%)
Moradian, 2015^26^	Randomized clinical trial	Breast cancer N=99	Rhodes Index of Nausea, Vomiting and Retching (INVR)	Music therapy	Nausea prevalence. Mean (SD) Vomiting prevalence. Mean (SD)			a. 4.31 (4.31) b. 1.38 (2.70)	a.3.0 (3.33) b.1.46 (3.29)
Substances for oral administration									
Ingersoll, 2010^27^	Randomized clinical trial	Different types of cancer except for head and neck cancer N=77	Rhodes Index of Nausea, Vomiting and Retching (INVR)	Flavonoid- rich adjunctive treatment (Concord grape juice)	Nausea and vomiting frequency Mean (SD)	1.6 (CI 95%: 0.6-2.6)	1.7 (CI 95%: 0.6-2.8)	1.6 (CI 95%: 0.3 2.9)	2.0 (CI 95%: 0.6-3.5)
Sanaati, 2016^28^	Randomized clinical trial	Breast cancer N= 65	Chemotherapy- induced nausea and vomiting (CINV)	a. Ginger capsules b. Chamomile capsules	a. Number of nausea. Mean difference (SD) b. Number of vomiting. Mean difference (SD)			Nausea: Ginger 1.5845 (0.57) a. Nausea: Chamomile 0.0769 (0.58) Vomiting: Ginger 0.108 (0.24) b. Vomiting: Chamomile 0.8394 (0.28)	
Manual therapies and reflexology									
Vanaki, 2016^29^	Randomized clinical trial	Breast cancer N= 108	Visual Analog Scale (VAS)	Therapeutic touch: Patterns of energy disturbance in the participant's body	Nausea duration. Mean (SD) Nausea frequency. Median (IQR)			a. 5.36 (2.17) b. 50.29	a. 10.81 (1.77) b. 31.44
Ozdelikara, 2017^30^	Randomized clinical trial	Breast cancer N= 60	Rhodes Index of Nausea, Vomiting and Retching (INVR)	Reflexology	a. Nausea and vomiting experience Mean (SD) b. Nausea and vomiting development. Mean (SD) c. Nausea and vomiting distress Mean (SD)	a. Nausea: 2.53 (2.80) Vomiting: 0.83 (1.57) Nausea: 1.83 (2.05) Vomiting: 0.56 (1.07) Nausea: 0.70 (0.83) c. Vomiting: 0.26 (0.52)	a. Nausea: 5.46(4.15) Vomiting: 3.83(4.29) b. Nausea: 3.70 (2.79) Vomiting: 2.40(2.82) c. Nausea: 1.76(1.38) Vomiting: 1.43(1.56)	Nausea: 2.06 (3.33) a. Vomiting: 0.96 (2.39) Nausea: 1.43 (2.35) b. Vomiting: 0.63(1.56) c. Nausea: 0.63(0.99) Vomiting: 0.33(0.84)	a. Nausea: 6.56(4.09) Vomiting: 4.0(3.29) b. Nausea: 4.40(2.82) Vomiting: 2.40(2.02) c. Nausea: 2.16(1.34) Vomiting: 1.60(1.35)
Acupressure									
Avci, 2016^31^	Randomized clinical trial	Myeloblastic Leukemia N= 90	Visual Analog Scale (VAS)	Acupressure, P6 point	Nausea severity Vomiting severity Number of nausea episodes Number of vomiting episodes	a. 3.3(0.8) b. 2.4(1.3) c. 5.5(0.8) d. 1.0(1.5)	a. 6.4 (0.6) b. 4.6 (0.9) c. 5.3 (1.3) d. 1.9 (0.6)	2.8(0.6) 1.4(1.3) 5.4 (0,8) 0.6 (0,5)	6.5(0.6) 4.6 (0.8) c. 6.6 (1.9) d. 2.2
Dibble, 2000^32^	Randomized clinical trial	Breast cancer N=17	Rhodes Index of Nausea, Vomiting and Retching (INVR)	Acupressure, P6 point	Nausea experience			2.83 (1.6)	3.00 (0.58)
Dibble, 2007^33^	Randomized clinical trial	Breast cancer N= 147	Rhodes Index of Nausea, Vomiting and Retching (INVR)	Acupressure, P6 point	Differences in the incidence of nausea between the experimental and control groups after the intervention.			RIN: c2 = 1.19, p = 1.23, p =	0.55; NRS: c2 = 0.55
Eghbali, 2016^34^	Randomized clinical trial	Breast cancer N=48	Morrow Assessment of Nausea and Emesis (MANE)	Auricular Acupressure	Nausea intensity. Mean (SD) Nausea frequency. Mean (SD) Vomiting intensity. Mean (SD) d. Vomiting frequency. Mean (SD)	a. 5.63 (3.98) b. 5.79 (6.4) c. 1.04 (1.71) d. 0.79 (1.33)	a. 3.71 (4.05) b. 3.54 (5.31) c. 2.29 (4.71) d. 2.08 (5.29)	2.08 (3.3) 1.85 (3.1) 0.79 (2.15) 0.54 (1.49)	7.54 (4.14) 6.85 (7.25) 3.71 (3.24) 2.06 (2.06)
Genç, 2013^35^	Quasi experimental	Lung, breast and cervical cancer N=64	Rhodes Index of Nausea, Vomiting and Retching (INVR)	Acupressure, P6 point	Nausea and vomiting experience. Z (P value)			Z=-3,88 P:0.0001 Experimental vs. Placebo: P<0.05	Z=-3.15 P: 0.0001
Genç, 2015^36^	Quasi experimental	Breast cancer N=64	Rhodes Index of Nausea, Vomiting and Retching (INVR)	Acupressure, P6 point	Nausea experience Vomiting experience Nausea occurrence Vomiting occurrence	4.71 (3.53) 3.96 (3.18) 3.28 (2.45) 2.56 (2.28)	a.5.57 (3.47) b.4.78 (2.85) c.3.84 (2.42) d.3.15(1.90)	a. 1.87 (2.60) b. 0.46 (1.64) c. 1.25 (1.77) d. 0.34 (1.12)	a. 4.75 (2.59) b. 0.31 (0.89) c. 3.12(1.73) d. 0.21 (0.60)
Molassiotis, 2007^37^	Randomized clinical trial	Breast cancer N=50	Rhodes Index of Nausea, Vomiting and Retching (INVR)	Acupressure, P6 point	Nausea experience Vomiting experience c. Nausea occurrence Vomiting occurrence Nausea distress Vomiting distress	a. 0.87 (2.2) b. 0.66 (2.6) c. 0.66 (1.6) d. 0.53 (2.1) e. 0.20 (0.6) f. 0.12 (0.5)	a. 1.46 (3.1) b. 0.94 (2.7) c. 2.16 (2.4) d. 0.66 (1.9) e. 0.55 (1.0) f. 0.28 (0.8)	a. 2.72 (3.1) b. 0.2 (0.5) c. 1.20 (2.6) d. 0.13 (0.5) e. 0.27 (0.6) f. 0.31 (0.4)	a. 2.5 (3.4) b. 0.5 (1.5) c. 1.94 (2.3) d. 0.22 (0.6) e. 0.55 (1.1) f. 0.67 (0.9)
Molassiotis, 2013^38^	Randomized clinical trial	Different types of cancer N=500	Rhodes Index of Nausea, Vomiting and Retching (INVR)	Acupressure, P6 point	Nausea and vomiting experience. Median (IQR) Nausea frequency N (%) Vomiting frequency. N (%)	a.1.0 (0.0-7.50) b.79 (63%) c.109 (87%)	a.1.43 (0.0-8.57) b.69 (59%) c.100 (85%)	a. 0.00 (0.0-9.86) b. 70 (78%) c. 71 (88%)	a. 1.14 (0.0-9.14) b. 50 (62%)
Molassiotis, 2014^39^	Randomized clinical trial	Different types of cancer N=334	Rhodes Index of Nausea, Vomiting and Retching (INVR)	Acupressure, P6 point	a. Nausea experience (range 0 to 12). Median (IQR)	1.0 (2.97 - 7.50)	1.43 (3.71 - 8.57)	0.00 (1.82 - 9.86)	1.14 (4.00- 9.14)
Shen, 2019^40^	Quasi experimental	Lung cancer N=70	Morrow Assessment of Nausea and Emesis (MANE)	Acupressure, P6 point	Nausea severity. Mean (SD) Vomiting severity. Mean (SD)	2.94 (0.8) 0.4 (0.1)	2.94 (0.9) 1.06 (1.4)	0.46 (0.7) 0.03 (0.2)	a.2.66 (0.8) b.0.8 (1.3)
Shin, 2004^41^	Randomized clinical trial	Gastric cancer N=40	Rhodes Index of Nausea, Vomiting and Retching (INVR)	Acupressure, P6 point	Severity. Mean (SD) Duration. Mean (SD) Frequency. Mean (SD)	a. 1.55 (3.42) b. 0.45 (1.36) c. 0.10 (0.45)	3.85 (6.38) 0.65 (1.46) c. 0.10 (0.45)	a. 6.05 (2.85) b. 1.70 (2.49) c. 0.30 (0.73)	a.9.55 (5.47) b. 4.25 (3.27) c. 0.90 (1.33)
Suh, 2012^42^	Randomized clinical trial	Breast cancer N=120	Rhodes Index of Nausea, Vomiting and Retching (INVR)	Acupressure, P6 point	a. Level of nausea and vomiting. Media (DE)	7.97 (5.1)	12.09(9.44)	3.12 (4.3)	9.17 (7.58)
Akhu-Zaheya, 2017^43^	Randomized clinical trial	Different types of cancer N=224	Functional Living Index Emesis (FLIE), Chemotherapy- induced nausea and vomiting (CINV)	Hologram bracelets	Vomiting frequency. Mean (SD) Nausea severity. Mean (SD) Vomiting severity. Mean (SD)	a. 0.26 (1.27) b. 1.00 (2.14) c. 0.44 (1.65)	a. 0.46 (1.46) b.1.09 (2.17) c. 0.72 (1.97)	a. 0.31(1.33) b. 1.82 (2.99) c. 0.59 (1.93)	a.0.59 (1.45) b. 2.91 (2.97) c. 1.28 (2.75)
Pearl, 1999^44^	Randomized clinical trial	Gynecologic cancer N=32	Not reported	Transcutaneous stimulation bracelet	Report of reduced vomiting intensity			71%	21%

**Table 3:** Non-pharmacological interventions: alopecia outcome

		Population and popula-tion					Outcome		
Author, year	Design	size N	Instrument	Intervention	Outcome		Before	After	
						Intervention	Control	Intervention	Control
Betticher, 2013^45^	Non-randomized controlled study	Different types of cancer N= 167	WHO alopecia grading (I: slight and regular hair loss, II: moderate hair loss, III: complete but reversible hair loss, IV: complete and irreversible hair loss)	Scalp cooling Paxman® PSC-2 machine (PAX)	Reduction of alopecia grades III and IV %			80%	78%
Giaccone, 1988^46^	Randomized clinical trial	Different types of cancer N= 39	Unclear. A 4-point grading scale is used: 0 no hair loss, 1 minimal hair loss (<25%), 2 moderate hair loss (25 50%), and 3 severe alopecia (>50%).	Hypothermia Cap (commercially available as Spenco Hypothermia Cap- Spenco Medical Corporation, Texas)	Hair loss (reduction of alopecia grade 3)			Grade 0:5 Grade 1:2 Grade 2:1 Grade 3:11	Grade 0:0 Grade 1:0 Grade 2:1 Grade 3:15
Kargar, 2011^47^	Non-randomized experiment	Unspecified cancers. N=63	WHO alopecia scale	Scalp cooling system	Hair loss (reduction of alopecia grades 3-4)	Grade 1-2: 24 (77.4%) Grade 3-4: 7 (22.6%)	Grade 1-2: 12 (38.7%) Grade 3-4: 19 (61.3%)	Grade 1-2: 15 (50%) Grade 3-4: 15 (50%)	Grade 1-2: 8 (25%) Grade 3-4: 24 (75%)
Macduff, 2003^48^	Randomized clinical trial	Breast cancer N=30	WHO alopecia scale	Cool cap	Hair loss (increase from grade 0 to 2)	Grades 0 a 2: 73%	Grades 0 a 2: 23%	Grades 0 a 2: 25%	Grades 0 a 2: 0%
Nangía, 2016^49^	Randomized clinical trial	Breast cancer N=182	CTCAE v. 4.0 grade 0 (No hair loss), grade 1 (Hair loss of <50% of normal but it does not require wearing a wig). Failure was defined as CTCAE v4.0 grade 2 (Hair loss of >50% normal and it requires wearing a wig).	Scalp cooling	Efficacy: success in hair preservation N (%)			N=95 Grade 0: 48 (50.5%) Grade 1: 5 (5.3%) Grade >2: 47 (49.5%)	N=47 Grade 0: 0 (0%) Grade 1: 0 (0%) Grade >2: 47 (100%)
Lemenage, 1997^50^	Randomized clinical trial	Different types of cancer N=98	WHO alopecia grading Grade 0: No hair loss Grade 1: Slight hair loss Grade 2: moderate hair loss Grade 3: complete but reversible hair loss Grade 4: complete and irreversible hair loss	Cool cap	Efficacy: Degree of alopecia less than 2 N (%)			Grades 0-1: 83 (85.60%)	Grades 2-4: 14 (14.4%)
Nolte, 2006^51^	Randomized clinical trial	Gynecologic cancer N=187	Breast cancer stem cells (BC SCs) (Secord & Jourand, 1953).	45-minute video featuring makeup techniques and suggestions for women's hairstyles and headpieces.	Body image perception			2.24 (0.61)	2.17 (0.53)
Rugo, 2017^52^	Randomized clinical trial	Breast cancer N=182	Dean scale	Scalp colling	Efficacy: success in hair preservation N (%)			67 (66.3%)	0 (0%)

**Table 4:** Non-pharmacological interventions: pain and neuropathy outcome

Author, year	Design	Popul	Instrumen	Interve	Outcom			Outcome	
		ation	t	ntion	e	Before			After
		and popula-tion size N				Intervention	Control	Intervention	Control
Nursing intervention program									
Molassiotis, 2009^17^	Randomized clinical trial	Colorectal and breast cancer N=164	CTCAE Toxicity Rating Scale (NIH/ NCI)	Home care nursing program	Toxicity grading Mean	NR	NR	2.9	6.3
Rustoen, 2014^53^	Randomized clinical trial	Different types of cancer with bone metastasis N=179	Care Needs Assessment (CNA)	Nursing program for pain management (PRO SELF)	Pain Mean -	3.6	3.7	2.7	3.1
De Wit, 1997^54^	Randomized clinical trial	Different types of cancer N=313	McGill Pain Questionnaire (MPQ-DLV)	Pain education program	Pain %	58.4	55.9	39.4	16.9
Muscle exercises									
Aghabati, 20 08^55^	Randomized clinical trial	Cancer patients N=90	Care Needs Assessment (CNA)	Therapeutic touch	Pain Mean	1.9	0.02	1	0
Miladinia, 2017^56^	Randomized clinical trial	Acute Leukemia N=64	Care Needs Assessment (CNA)	Slow-Stroke Back Massage (SSBM)	Pain Mean	6.5	6	4.8	6.3
Dhawan, 2020^57^	Randomized clinical trial	Different types of cancer N=45	Chemotherapy- induced peripheral neuropathy (CIPN)	Muscle strengthening exercises	Neuropathy Mean	132.5	129.3	83.1	140.8
Self-affirmation									
Yildirim, 20 1 7^58^	Randomized clinical trial	Different types of cancer N=140	Edmonton Symptom Assessment System (ESAS)	Self-affirmation	Pain Mean	0.66	1.31	0.09	2.03
Given, 2015^59^	Randomized clinical trial	Different types of cancer N=113	Symptom experience scale	Supportive care	Pain n (%)/mean	29(69)/7.3	30(63)/6.8	19(54)/3.3	25(58)/4.4
Foot bath									
Park, 2015^60^	Quasi experimental	Colorectal and gastric cancer N=48	CTCAE Toxicity Rating Scale (NIH/ NCI)	Foot bath	Neurotoxicity grades 2 and 3 n (%)	24(100)	24(100)	20(83)	21(87.5)
Neural gliding									
Andersen, 2020^61^	Randomized clinical trial	Breast cancer N=61	Disability of the Arm, Shoulder and Hand (DASH) questionnaire	Nerve gliding exercises	Neuropathy. Mean	44.1	44.8	40.6	45.9
Acupuncture									
Zhi, 2022^62^	Randomized clinical trial	Different types of cancer N=63	Quantitative Sensory Testing (QST)	Acupuncture	Thermal neuropathy n/mean	21/46.31	19/46.31	17/47.12	16/46.96
Arslan, 2020^63^	Randomized clinical trial	Colorectal and gastric cancer. N=60	CTCAE Toxicity Rating Scale (NIH/ NCI)	Henna application	Neuropathy Mean	65	67.9	40.9	68.4
Greenlee, 2016^64^	Randomized clinical trial	Breast cancer N=63	Net Promoter Score de 4 (NPS-4 score)	Acupuncture	Neuropathy Mean	16.8	35.2	7.9	18

**Table 5:** Non-pharmacological Interventions: Sleep Disorders

Author, year	Design	Population and popula-tion	Instrument	Intervention	Outcome	Outcome			
		size N				Before		After	
						Intervention	Control	Intervention	Control
Acupressure									
Tsao, 2019^65^	Quasi experimental	Ovarian cancer N=60	PSQI- Pittsburgh Sleep Quality Index	Acupressure	Sleep quality Mean	2.5	2.24	2.4	4.05
Kuo, 2018^66^	Randomized clinical trial	Ovarian cancer N=40	PSQI- Pittsburgh Sleep Quality Index	Acupressure	Sleep quality Mean	13.2	12.25	4.21	12.75
Telephone follow-up programs									
Barsevick, 2010^11^	Randomized clinical trial	Different types of cancer N=276	PSQI- Pittsburgh Sleep Quality Index	Telephone follow ups and education	Sleep quality Mean	8.01	7.83	7.96	8.24
Physical exercise programs									
Coleman, 2012^67^	Randomized clinical trial	Multiple myeloma N=187	Actigraphy*	Physical exercise program	Sleep quality Mean	79.7	81.39	77.79	76,57
Foot bathing									
Yang, 2010^68^	Randomized clinical trial	Gynecologic cancers N=50	Verran y Snyder-Halpern Sleep Scale	Warm-water footbath	Sleep quality Mean	805.5	743	944.9	763.2
Movement and relaxation practices									
Chuang, 2017^69^	Randomized clinical trial	Non-Hodgkin lymphoma N=96	Verran y Snyder-Halpern Sleep Scale	Practice of Chan- Chuang qigong	Sleep quality Mean	657	79.7	922.9	77.19
Yang, 2021^70^	Cohort	Ovarian cancer N=389	PSQI- Pittsburgh Sleep Quality Index	Exercise and cognitive behavioral therapy	Sleep quality Mean	13.94	14.76	14.29	14.37
Reich, 2015^71^	Randomized clinical trial	Breast cancer N=79	PSQI- Pittsburgh Sleep Quality Index	Mindfulness	Sleep quality Mean	7.97	8.39	6.91	6.91
Baraz, 2017^56^	Randomized clinical trial	Acute leukemia N=64	PSQI- Pittsburgh Sleep Quality Index	Slow-Stroke Back Massage on Symptom (SSBM)	Sleep quality Mean	12.23	9.7	12.1	12.37

*Actigraphy: An instrument used to monitor sleep and wakefulness patterns.

**Table 6:** Non-pharmacological Interventions: Neutropenia

Author, year	Design	Population and	Instrument	Intervention	Outcome	Outcome			
						Before		After	
		population size N				Intervention	Control	Intervention	Control
Mei Ling Yeh,	Quasi-	Breast cancer	SYSMEX9000	Chan-Chuang qi-gong	WBC count	1.955 |Æ	1.955 |Æ	> 416.25 |Æ	> 810.57 |1L
2006^72^	experimental	N: 67	automatic blood	therapy	Hemoglobin	11.42 g/dL	11.32 g/dL	< 0.27 g/dL	< 0.43g/dL
			analyzer		Platelets	189,500 |Æ	194,523 |Æ	> 92,531.25|1L	> 67,057.14 |1L
Chuang TY,	Randomized	Non-	Beckman	Chan-Chuang qi-gong	WBC count	4,731.46 |Æ (SD 2,074.34 |1L)	5,482.29 |Æ (SD 3,460.63 |1L)	6,478.33 |1L (SD 4,222.05 |1L)	4,150.42 |1L (SD 2,142.67 |1L)
2017^69^	clinical trial	Hodgkin lymphoma N:100	automatic blood analyzer	therapy	Hemoglobin Platelets	11.64 g/dl (SD 2.03 g/dL) 173,479.17(SD 96,707.49 |1L)	11.39 g/dL(SD 2.03 g/dL) 200,645.83 |1L (94,867.32 |1L)	11.97 g/dL (SD 2.06 g/dL) 177,395.83 |1L (SD 80,056.29 |1L)	11.07 g/dL (SD 2.15 g/dL) 179,250.00 |1L (SD 80,795.38 |1L)

## Discussion

This review described non-pharmacological interventions for controlling the primary side effects of chemotherapy with a high degree of heterogeneity and internal validity among the studies. This is consistent with some studies stating that non-pharmacological interventions are complementary to medical treatments; however, they emphasize the lack of valid evidence to present the effect of these interventions as complementary to pharmacological treatments^73^^, ^^74^.

The review described several types of non-pharmacological interventions to address the side effects of chemotherapy. These interventions include education and exercise programs, hypothermia devices, acupressure techniques, music therapy, traditional Chinese medicine techniques, relaxation techniques, foot baths, and transcutaneous electrical nerve stimulation^75^^, ^^76^.

Nurse-led home-based patient education programs are designed to manage symptoms. These non- pharmacological interventions have shown measurable differences in pain levels before and after the intervention^54^^, ^^59^. A need was identified to standardize educational programs and to know the content and indicators for pain assessment^26^^, ^^76^^, ^^79^. However, for patients with multiple symptoms, these processes should be accompanied by psychological support and strengthening of mental health to ensure beneficial application and results in the control of the symptoms.

Holistic medical systems such as acupressure have been studied extensively. This review found that acupressure consistently reduced nausea and vomiting compared to standard care in all measurements^36^. This result is consistent with the study by Lee A et al. ^78^, who conducted a review and found that acupressure at the P6 point has a moderate effect compared to placebo, although the studies have limitations in terms of variation in effects and methodological quality. However, when comparing acupressure with antiemetics, no difference in the incidence of nausea and vomiting was observed. Therefore, it can be concluded that the available evidence may support a combined therapy of P6 point stimulation and antiemetic drugs rather than drug prophylaxis alone and that further high-quality trials are needed^76^^, ^^79^^).^

Manipulative and body-based practices, such as muscle relaxation therapies, reflexology, and therapeutic touch, along with sensory intervention techniques like music therapy and guided imagery, have been described and evaluated with positive effects^80^^, ^^81^; however, the reported studies record wide variability of populations, techniques, and study periods regarding outcomes such as pain, nausea and vomiting^76^^, ^^81^. The main limitation of these studies was the lack of control for confounding factors, such as the use of medications and other therapies and individual perception of the symptom.

It is important to consider that these types of studies are valuable in building the body of evidence that will later support evidence-based recommendations^82^. The literature consistently states that acupressure is a complementary technique and does not replace traditional treatment^79^. The reported studies agree that environmental factors and the use of patients' unreported therapies limit the evaluation of interventions; hence, there is a need to identify what type of interventions patients are conducting.

The immune system's vulnerability to opportunistic infections and the extended duration of treatment make neutropenia a priority in evaluating non-pharmacological interventions. Chan-Chuang qigong therapy has been evaluated in people diagnosed with cancer^69^^, ^^72^ and showed an increase in white blood cell count before and after the intervention. However, variables such as time, comorbidities, and treatments must be controlled to estimate the true effect of this intervention.

Alopecia is one of the secondary symptoms that compromise biological, psychological, emotional, and social aspects, affecting the health status of people who suffer from it and is increasingly becoming a priority outcome for the well-being of patients^83^^, ^^84^. Video tutorials for makeup, wig styling, and scalp cooling are techniques that have been increasingly reported in recent years to mitigate these effects and improve the quality of life for patients. There is a need to further clarify alopecia measurement strategies with validated scales for different populations.

This review included observational and experimental studies, giving a broad overview of the interventions reviewed. These results suggest some implications for clinical practice and future research. First, each of these interventions and their results should be considered with caution since the representativeness of the populations and the standardization of the techniques used can only be generalized to patients with characteristics similar to those studied in the included studies. Secondly, for research purposes, it is highly recommended that future reviews focus on interventions by symptom clusters^85^. The search strategies used in this review enabled us to capture the broadest selection of relevant literature according to the side effects of chemotherapy using distinct search terms. The included studies showed low methodological quality and evidence that interventions could have a real effect on controlling various symptoms, as evidenced by acupressure on symptoms such as nausea and vomiting, sleep disorders, pain, and neuropathy. The findings of this review highlight the gaps in the available literature and emphasize the importance of further documenting the effect of non-pharmacological interventions on chemotherapy side effects.

## Conclusion

Prioritizing side effects for patients guides care plans for individuals. Non-pharmacological interventions such as acupressure, Chinese therapies such as Chan-Chuang qigong, muscle relaxation therapies, and nursing intervention programs have been evaluated and described with evidence for nausea and vomiting, pain and neuropathy, sleep disorders, alopecia, neutropenia, and anorexia. However, there is still high variability in the type of intervention, outcomes measuring, and lack of statistical power, making it difficult to estimate the effects of these interventions. Research with methodological rigor and standardization of these interventions is needed to validate their effects on these outcomes.
